# Fe_3_O_4_@chitosan Microspheres Coating as Cytoprotective Exoskeletons for the Enhanced Production of Butyric Acid With *Clostridium tyrobutyricum* Under Acid Stress

**DOI:** 10.3389/fbioe.2020.00449

**Published:** 2020-05-15

**Authors:** Tingting Liu, Cheng Jiang, Liying Zhu, Ling Jiang, He Huang

**Affiliations:** ^1^College of Biotechnology and Pharmaceutical Engineering, Nanjing Tech University, Nanjing, China; ^2^College of Food Science and Light Industry, Nanjing Tech University, Nanjing, China; ^3^College of Chemical and Molecular Engineering, Nanjing Tech University, Nanjing, China; ^4^College of Pharmaceutical Science, Nanjing Tech University, Nanjing, China

**Keywords:** *Clostridium tyrobutyricum*, butyric acid, Fe_3_O_4_@chitosan, protective exoskeleton, acid stress, repeated-batch fermentation

## Abstract

The introduction of inorganic nano-materials may endow microbial cells with unique new features, including greater resistance to adverse abiotic stress. The aim of this work was to enhance the acid tolerance of *Clostridium tyrobutyricum* ATCC 25755 by coating cells with self-assembled Fe_3_O_4_@chitosan (Fe_3_O_4_@CS) microspheres, and thereby increase the production of butyric acid. The optimal coating efficiency of 81.19% was obtained by systematically optimizing the three operational parameters temperature, rpm and mass ratio, which were determined to be 37°C, 80 rpm and 1:2, respectively. Physicochemical characterization was used to assess the superparamagnetism, thermostability and subsize of Fe_3_O_4_@CS attached to the cells. Compared to free cells, *C. tyrobutyricum* coated with Fe_3_O_4_@CS (CtFC) exhibited stronger acid tolerance at low pH. At a pH of 4 or 5, the levels of ROS, MDA, LDH, and SOD caused by the acid environment in free cells were significant higher than in CtFC. Moreover, without adding NaOH, CtFC fermentation showed a higher butyric acid titer (37.60 vs. 31.56 g/L) compared to free-cell fermentation. At the same time, an average butyric acid yield of 0.46 g/g in each repeated-batch fermentation was also obtained by taking advantage of the biocatalyst’s reusability and convenient separation from the fermentation broth via an external magnetic force. Overall, the developed CtFC illustrates a new paradigm for developing an economical and reusable biocatalyst for industrial application in butyric acid production.

## Introduction

Butyric acid is well-known as a typical short-chain fatty acid (SCFA), and is widely applied in various products including food, perfumery, pharmaceuticals and animal feed supplements (Jiang et al., 2018; Oh et al., 2019). Although current industrial butyric acid production is dominated by chemical synthesis, the striving for sustainable development inspired increasing research on bio-based production of butyric acid from renewable resources (Lee et al., 2016). In order to fulfill high-efficiency and economic production of bio-based butyric acid, research efforts have focused on metabolic engineering (Jang et al., 2014; Suo et al., 2018), process development (Jiang et al., 2011; Luo et al., 2020) and low-cost biomass feedstocks utilization (Chi et al., 2018; Xiao et al., 2018). *Clostridium tyrobutyricum* is a Gram-positive, strictly anaerobic and spore-forming *Bacillus* that produces butyric acid from a number of substrates, including glucose, lactose, xylose, and glycerol (Jiang et al., 2009; Fu et al., 2017). It is widely considered the major butyrate production strain, offering final concentreation as high as 86.9 g/L (Jiang et al., 2011). However, the industrial development of biotechnological butyrate production is seriously restrained by the strains’ sensitivity to oxygen stress, acid stress, and substrate toxicity, as well as the difficult product separation. To address this challenge, preliminary studies in synthetic biology and genetic engineering have been conducted to develop elaborate mechanisms and enhance cellular adaptive responses to abiotic stresses (Liu et al., 2017; Wu et al., 2017). However, these approaches still face significant limitations due to complexity, epigenetics, mutations, and so on (Endy, 2005; Purnick and Weiss, 2009; Kim et al., 2016). As a result, a widely applicable strategy for strengthening the adaptability of living cells without relying on genetic engineering is highly anticipated (Jiang et al., 2012). Some microorganisms such as *Bacillus subtilis* form an endospore coating, also defined as a robust multilayer shell, to protect the bacterial genome under stress conditions (McKenney et al., 2013). The biological self-protection mechanisms of *B. subtilis* inspired artificial strategies for preparing mechanically durable coatings on the surface of cells. Recently, several studies reported that some polymers, inorganic nanoparticles, and MOFs could be used to produce protective cytoprotective exoskeletons against ambient stress. For instance, crystallizing the MOF material ZIF-8 on the surface of *Saccharomyces cerevisiae* resulted in a protective shell that can increase cell viability under adverse conditions (Liang et al., 2016). A combination of layer-by-layer self-assembly with biomimetic silicification was successfully applied to form silica coats on living cells without damage (Yang et al., 2009). Similarly, vesicles were developed to serve as protective shells, which served as living modules in certain synthetic cells (Elani et al., 2018). The construction of organism-material hybrids spawned the development of new features, which may lead to superior resistance to stress stimuli.

Magnetic nanomaterials based on Fe_3_O_4_, such as nanoparticles with a high specific surface area, good biocompatibility, high affinity and low mass transfer resistance, have been applied as a carrier for the immobilization of enzymes and drug delivery (Atacan and Özacar, 2015). As a hydrophilic macromolecular biomaterial, chitosan was used as an adhesive substance for coating cells with Fe_3_O_4_ nanoparticles to further enhance the biocompatibility of a prepared Fe_3_O_4_@chitosan (Fe_3_O_4_@CS) compound (Liu et al., 2011). The functional -NH_2_ and -OH groups of chitosan promote the stable covalent binding of the Fe_3_O_4_@CS compound onto the surface of bacteria (Liu et al., 2011). Moreover, studies have reported that the Fe_3_O_4_@CS compound can create a better microenvironment around cells (Vinila et al., 2014; Xiao et al., 2017). Based on these characteristics, the Fe_3_O_4_@CS compound deemed to have promising prospects as cytoprotective exoskeletons for living organisms against abiotic stimuli.

In our study, a protective coating of *C. tyrobutyricum* by the Fe_3_O_4_@CS (termed as CtFC) compound was prepared by co-precipitation via the cross-linking reaction of glutaraldehyde. To obtain the optimal coating efficiency on *C. tyrobutyricum*, an L_9_ (3^3^) orthogonal array with temperature, rpm and the mass ratio of the Fe_3_O_4_@CS to the DCW as three operational parameters was used. The oxidative and cellular damage of free cells and CtFC at low pH was determined to assess the protective effect of the Fe_3_O_4_@CS shell on *C. tyrobutyricum* cells in an acidic environment. The reusability and separation of CtFC from the fermentation broth may facilitate the development of novel biocatalysts for the high-yield production of butyric acid.

## Materials and Methods

### Reagents

Ferric chloride, ferrous chloride, glutaraldehyde (25%) and Span-80 were purchased from Xilong Chemical Company (Guangdong, China). Chitosan and acetic acid were purchased from Sinopharm Chemical Reagent, Co. (Shanghai, China). Petroleum ether, ethanol (95%), ammonium hydroxide and liquid paraffin were procured from Myrell Chemical Technology (Shanghai, China). Unless otherwise specified, all chemical reagents were of analytical grade.

### Strains, Media, and Culture Conditions

*Clostridium tyrobutyricum* ATCC 25755 was purchased from Guangdong culture collection center (Collection number: GIM 1.262), and cultured anaerobically at 37°C in Reinforced Clostridial Medium (RCM) containing (per liter): 10 g tryptone (OXOID, United Kingdom), 10 g beef extract (Hopebio, China), 5 g NaCl, 3 g yeast extract (OXOID, United Kingdom), 3 g anhydrous sodium acetate, and 0.5 g L-cysteine. Glucose were prepared as the carbon source in a separate anaerobic solution and added to the basal medium after autoclaving. Oxygen was removed by sparing with high-purity nitrogen (99.99%) and confirmed by adding resazurin to a final concentration of 0.05%.

### Synthesis of Magnetic Fe_3_O_4_ Nanoparticles

The procedure for preparing Fe_3_O_4_ nanoparticles was based on a modified co-precipitation method (Liu et al., 2011). First, 5.459 g ferric chloride and 2.060 g ferrous chloride were dissolved in 100 mL of deionized water in a three-necked flask (250 mL) and stirred under N_2_ at 800 rpm and 60°C for 1 h to allow the synthesis of nanoparticles particles in a magnetic force oil bath. Ammonium hydroxide was added dropwise to adjust the pH of the solution to between 9 and 10 during stirring. Magnetic precipitate was collected and isolated using a strong magnet (60 mm × 20 mm × 10 mm), washed with ethanol several times until reaching a neutral pH value, and dried at 50°C in an electric thermostatic drying oven. Fe_3_O_4_ nanoparticles were collected by grinding and used for further experiments.

### Preparation of Magnetic Fe_3_O_4_@CS Nanoparticles

Synthetic Fe_3_O_4_ (0.4 g) was added to a chitosan-acetic acid solution (0.28 g chitosan dissolved in 15 mL of 5% acetic acid solution) to achieve a molar ratio of Fe_3_O_4_: chitosan of 1:1, and dispersed by ultrasound for 30 min. Liquid paraffin (60 mL) and Span-80 (1 mL) were added into the mixture of Fe_3_O_4_@CS to prepare an emulsion, which was subsequently stirred for 30 min at 40°C. Then, 2 mL of 25% glutaraldehyde solution was added, after which the reaction solution was heated and stirred vigorously at 60°C for 3 h. The Fe_3_O_4_@CS nanoparticles were obtained from the reaction mixture by lyophilization at −55°C in a FDU-1200 freeze-dryer (EYELA, Tokyo) for 48 h, followed by washing three times with petroleum ether, deionized water and ethanol, respectively.

### Optimization of the Formation of the Fe_3_O_4_@CS Exoskeletons Coating on the Surface of *C. tyrobutyricum* Cells

The Fe_3_O_4_@CS nanoparticles were sterilized by soaking in deionized water. A suspension of *C. tyrobutyricum* was prepared by suspending cells cultured under anaerobic conditions at 37°C for 12 h in 100 mL of sterile saline. To optimize the efficiency of the Fe_3_O_4_@CS cell coating, an orthogonal test was performed (Wei et al., 2013). An L_9_ (3^3^) orthogonal array with temperature, rpm and the mass ratio of the Fe_3_O_4_@CS to the DCW as three operational parameters was used. The coating efficiency (*C*%) was determined and calculated according to the equation:

(1) $$C\%=\frac{A_{0}-A_{i}}{A_{0}}\times 100$$

where *A*_0_ is the initial value of the optical density (OD) at 600 nm of the cell suspension, and *A*_i_ is the final OD_600_ of the cell suspension after CtFC was collected using a magnetic field. The optimized surface coating parameters were assessed on the basis of the optimal coating efficiency.

### Physical Characterization

The synthetic Fe_3_O_4_, Fe_3_O_4_@CS, and CtFC obtained under the optimal surface coating parameters were selected for physical characterization. Scanning electron microscopy images and energy dispersive spectrometry (EDS) of samples were recorded on a SU8020 SEM (Hitachi, Japan) at an accelerating voltage of 15.0 kV. A vibrating sample magnetometer (VSM; model 7410, United States) was used to examine the magnetic properties with a magnetic field from 30 to −30 kOe at room temperature. Thermogravimetric analysis (TGA) and differential scanning calorimetry (DSC) were performed on a TGA/DSC1/1600HT analyzer (Mettler-Toledo, Switzerland) in air to determine the thermal stability of the samples. Fourier transform infrared spectroscopy (FTIR) spectra of the samples were recorded on a Spectrum GX instrument (Perkin-Elmer, United States) in the wave number range of 4000–500 cm^–1^ at RT. The powder X-ray diffraction patterns were recorded on a X’pert Pro MPD diffractometer (PANalytical, Netherlands) to study the crystal structure of the samples. The crystallite size of Fe_3_O_4_ was calculated in Jade software, using the Scherrer equation:

(2) $$D=\frac{K\,\gamma }{B\,\text{cos}\,\theta }$$

where the Scherrer constant K is 0.89, B is the full width at half maximum of the sharp peaks, γ is the wavelength of X-ray diffraction, and θ is the measured diffraction angle (Vinila et al., 2014).

### Effects of Environmental Acidity on the Growth Curves, Cell Viability, and Membrane Potential of Free Cells and CtFC

*Clostridium tyrobutyricum* cells coated with Fe_3_O_4_@CS were incubated anaerobically at different pH values (3.0, 4.0, 5.0, 6.0, 7.0) at 37°C for 12 h. Free cells were used as the control group. The growth performance of the experimental group and control group was evaluated by measuring the cell density. Cell viability and membrane potential were determined using a Mitochondrial Membrane Potential Detection Kit (C1071; Beyotime, Shanghai, China). The cells were harvested by centrifugation for 5 min at 1,000 *g* and resuspended in 50 mM phosphate buffered saline (PBS, pH 7.0). A total of 50,000 cells collected by centrifugation were resuspended with 188 μL Annexin V-FITC. The mixed solution was prepared by adding 2 μL Mito-Tracker Red CMXRos, 5 μL Annexin V-FITC, incubated for 30 min at 25°C, and then placed in an ice bath. Finally, sample smears were visualized under a Leica DMi8 fluorescence microscope (Leica Microsystems, Germany). The whole process was carried out in the dark by using aluminum foil.

### Effects of Environmental Acidity on ROS, SOD Activity, MDA and LDH Content in Free Cells and CtFC

Before the indicators were measured, free cells and CtFC cultured for 12 h were preprocessed by removing the culture supernatant by centrifugation at 12,000 *g* for 2 min at 4°C, and washed with PBS (pH 7.0) twice. Superoxide dismutase (SOD) activity was measured using the xanthine oxidase/nitroblue tetrazolium (NBT) detection method (Sun et al., 2016). SOD activity was reflected by the inhibition of the reduction of NTB by superoxide radicals from the xanthine-xanthine oxidase reaction system. The harvested cells were suspended in 1 mL of extracting solution per 5 × 10^6^ cells and homogenized using an ultrasonicator (HN-150Y; Hanuo Instruments, Shanghai, China) at 200 W for 6 min (ultrasonication time: 3 s; rest time: 10 s). The cell supernatants of the control group and experimental group were collected by centrifugation at 8,000 *g* for 10 min at 4°C, and the absorbance at 560 nm was measured using a Multiskan Sky microplate reader (Thermo Fisher Scientific, United States).

The lactate dehydrogenase (LDH) content used to evaluate the membrane integrity was measured via the oxidation of lactic acid into pyruvic acid. The reaction mixture was incubated for 30 min at 25°C and then the absorbance at 450 nm was measured.

The concentration of malondialdehyde (MDA) was determined using the modified method of Choudhary et al. (2007). The homogenate prepared by adding 1% trichloroacetic acid o the bacterial cells was cleared by centrifugation at 10,000 *g* for 10 min. The obtained supernatant was heated at 100°C for 30 min with 5% thiobarbituric acid and then cooled in an ice-bath. Then, the mixture was centrifuged at 5,000 *g* for 5 min, and the absorbance at 532 and 600 nm was recorded.

The ROS levels were measured using the method of Hong et al. (2009). The cells were resuspended in PBS containing 10 mM DCFH-DA, incubated in the dark for 60 min at 25°C, and then washed twice with PBS. The relative SOD, LDH, MDA and ROS levels were calculated according to the following formula:

(3) $$R\,e\,l\,a\,t\,i\,v\,e\,l\,e\,v\,e\,l=\frac{Q\,1}{Q\,2}\times 100\%$$

where *Q*1 and *Q*2 are the mean absorbances of the control group and experimental group, or the fluorescence intensity values in the ROS level measurement.

### Butyric Acid Fermentation

Batch fermentation of butyric acid using CtFC was carried out in a 2-L NBS fermentation tank with 1 L of culture medium with 80 g/L glucose as the carbon source. To start the fermentation, 50 mL of cell suspension in serum bottle was inoculated to the fermentation tank until the cellular growth entry into the stationary phase. The pre-calculated quantity of nanocomposite Fe_3_O_4_@CS was then added into the bioreactor to form the CtFC compound. Next, the CtFC was separated from the fermentation broth and washed with PBS three times using an external magnetic field. The fresh liquid medium with the initial pH was added to the anaerobic bioreactor to regenerate CtFC under the same culture conditions to proceed the fermentation of butyric acid. In the repeated batch mode, the fresh liquid culture was replaced several times whenever the glucose concentration in the fermentation broth was close to zero. The fermentation broth collected during several fermentation processes was taken at regular intervals for further analysis.

### Statistical Analysis

Cell density was evaluated by measuring the optical density (OD) at 600 nm using an SP-752 spectrophotometer (Shanghai Spectrum, China). The concentrations of butyric acid and acetic acid were determined by high performance liquid chromatography (HPLC) on a Model L-2000 system (Hitachi, Japan) equipped with an Aminex HPX-87H organic acid analysis column (100 mm × 7.8 mm, Biorad, Marnes-la-Coquette, France), with 2.5 mM H_2_SO_4_ as the mobile phase at a flow rate of 0.6 mL/min. The analytes were detected using with a UV detector at 210 nm (G1314A, Agilent HPLC 1100 series).

## Results and Discussion

### Optimizing the Cell Coating Efficiency of Fe_3_O_4_@CS

**FIGURE 1:**
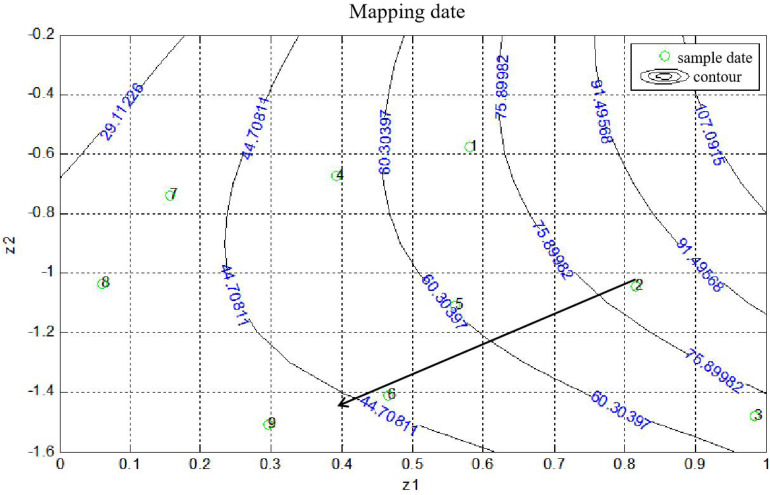
Contours on the mapping plane for the coating efficiency of Fe_3_O_4_@CS microspheres on *C. tyrobutyricum*. The green circles and numbers represented sample data, full line represented contours, long arrow represented the direction of data optimizing.

The statistical analysis and quantitative evaluation of the effect of the different factors on the coating efficiency was conducted based on an orthogonal experimental design. The three crucial parameters that affect the coating efficiency are listed in Table 1. A total of nine group experiments were performed and the respective results are shown in Table 2. According to the results of the orthogonal experiment, optimal coating conditions were centered around a temperature of 37°C, and the optimal coating efficiency was 80.61%, which exceeded the published cell coating efficiency of carboxyl Fe_3_O_4_ (Huang et al., 2018). These result implied that the specified temperature parameter was the most influential factor, which was consistent with a study by Guo et al. (2017), who found that temperature was conducive to the diffusion of Fe_3_O_4_ particles. In addition, a decreased temperature led to a decrease of coating efficiency. Relatively superior coating efficiency was observed at a stirring speed of 80 rpm and a mass ratio of Fe_3_O_4_@CS to the DCW of 1:2. Based on these data, we concluded that a higher temperature and a lower mass ratio of Fe_3_O_4_@CS to DCW improved the coating efficiency. The range analysis was performed to reflect the significance levels of the three influencing factors on the cell coating efficiency (Yu et al., 2008). Table 2 also shows that the significance levels of these three influencing parameters was in the order temperature (0.3584) > speed (0.0795) > mass ratio (0.0560). The intelligent visualization software (version 1.0, China), which utilizes dimension-reduction mapping to analyze the experimental results, was used to further explore the optimization direction and identify the optimal coating conditions based on the contour of the objective function (Yan and Bogle, 2007). As can be seen in Figure 1, nine experimental groups were marked by the nine points in the mapping diagram, and the black line represents the contour for the coating efficiency of each group (Song et al., 2016). The optimal point can be mapped inversely to the original multidimensional space by utilizing an inversion mapping algorithm and represented in terms of ratio data. Based on this, taking points 2 and 6 as references and using a step size of 2, the predicted optimal point marked by a full red solid rim was acquired through extrapolation in the direction of the arrow. The optimal point was encompassed a temperature of 37°C, a speed of 80 rpm and a mass ratio of Fe_3_O_4_@CS to the DCW of 1:2. To confirm the reliability of the optimal point, a verification experiment was conducted, and the results were in excellent agreement with the actual data (81.19 vs. 80.61%). Moreover, the *p-*value (60.20) and *F-*value (0.016) in the variance analysis (Table 3) confirmed that temperature was the most influential factor. The 98.49% value of R-sq in the fitting degree of the model (Table 4) underscored the high degree of agreement with the measured data.

**TABLE 1:** Factors and parameters of orthogonal experimental design.

**Factor**	**A/Temperature (°C)**	**B/Speed (rpm)**	**C/Proportion (g/g/L)^a^**
1	37	60	2:1
2	20	80	1:1
3	4	100	1:2

*^*a*^The mass proportion of Fe_3_O_4_@chitosan (g): DCW (g/L).*

**TABLE 2:** The design of L_9_(3^3^) orthogonal experiment and range analysis of influencing factors on coating efficiency.

**Factor number**	**Temperature (°C)**	**Speed (rpm)**	**Proportion (g/g)**	**Coating efficiency (%)**
1	37	60	2:1	72.34
2	37	80	1:1	80.61
3	37	100	1:2	69.70
4	20	60	1:1	52.94
5	20	80	1:2	61.84
6	20	100	2:1	46.81
7	4	60	1:2	40.61
8	4	80	2:1	36.21
9	4	100	1:1	38.30
K1	2.227	1.659	1.554	Total: 4.994
K2	1.616	1.787	1.719	
K3	1.151	1.548	1.722	
K①	0.7422	0.5530	0.5179	
K②	0.5386	0.5955	0.5728	
K③	0.3837	0.5160	0.5738	
R	0.3584	0.0795	0.0560	
Row rank	1	2	3	

**TABLE 3:** The variance analysis results.

**Sources**	**DOF^a^**	**Adj SS**	**Adj MS**	***F*-value**	***p*-value**	**Significance**
Temperature	2	0.194	0.097	60.20	0.016	*
Speed	2	0.0095	0.00475	2.95	0.253	
Proportion	2	0.00616	0.00308	1.91	0.344	
Error	2	0.00322	0.00161			
Total	8	0.213				

^*a*^Degrees of freedom. *Temperature is the most influential factor.

**TABLE 4:** The fitting degree of the model.

**S**	**R-sq**	**R-sq (adjustment)**
0.0401	98.49%	93.95%

### Synthesis and Characterization of Fe_3_O_4_, Fe_3_O_4_@CS, and CtFC

**FIGURE 2:**
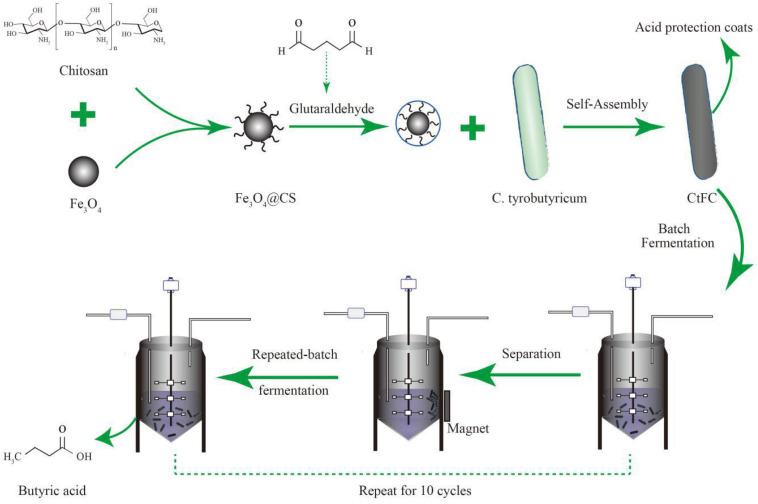
Schematic illustration of the generation process of CtFC and the repeated-batch fermentation of butyric acid by utilizing CtFC.

**FIGURE 3:**
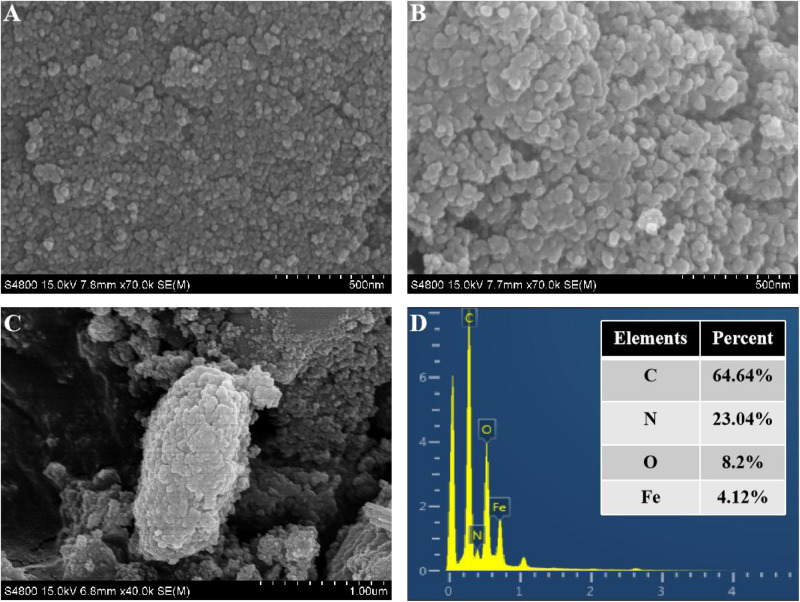
SEM images of naked Fe_3_O_4_ nanoparticles **(A)**, Fe_3_O_4_@CS microspheres **(B)**, and CTc **(C)**. EDS spectra of CTc **(D)**.

**FIGURE 4:**
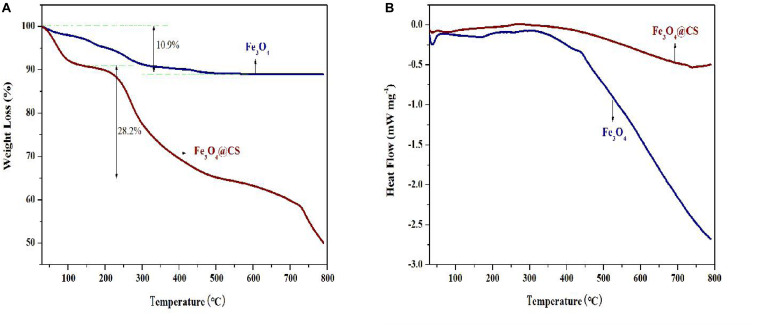
TGA **(A)** and DSC **(B)** curves of Fe_3_O_4_ particles and Fe_3_O_4_@CS.

**FIGURE 5:**
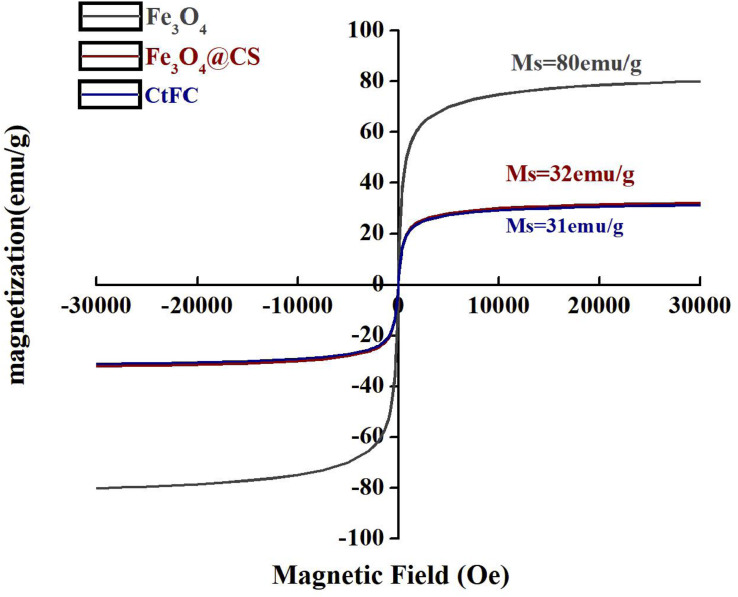
Magnetic hysteresis loops of Fe_3_O_4_ nano particles, Fe_3_O_4_@CS and CtFC.

**FIGURE 6:**
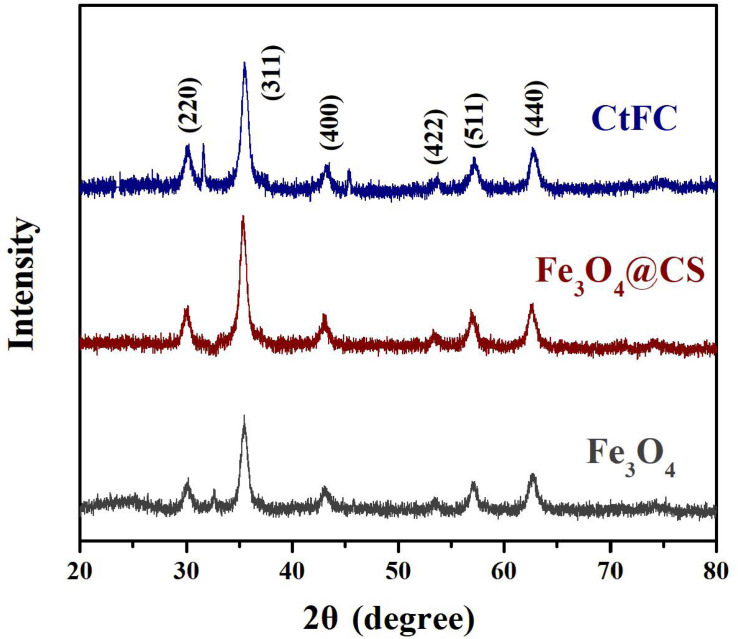
XRD pattern of Fe_3_O_4_ particles, Fe_3_O_4_@CS microspheres and CtFC.

**FIGURE 7:**
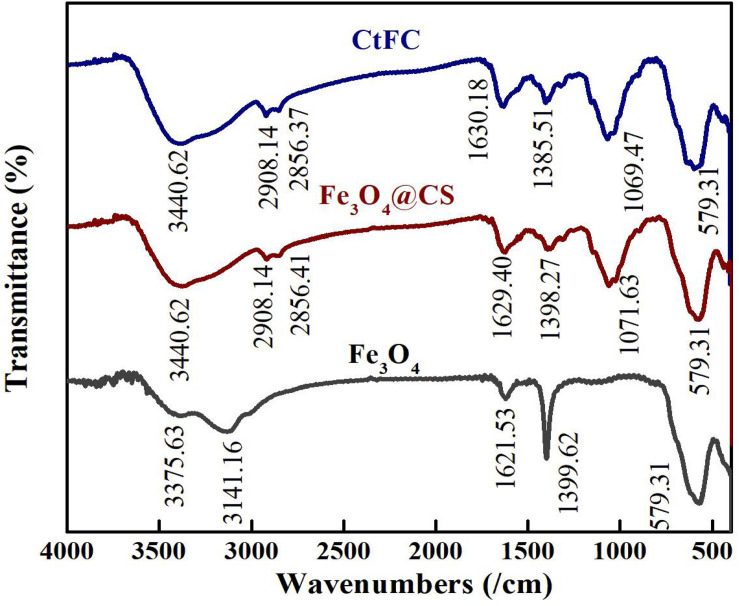
FT-IR spectra of Fe_3_O_4_ particles, Fe_3_O_4_@CS microspheres and CtFC.

CtFC obtained under the optimal coating conditions was selected for physical characterization. Figure 2 illustrates that CtFC comprises three components: the Fe_3_O_4_ particles for magnetic separation, chitosan for cell coating, and *C. tyrobutyricum* for butyric acid production. Abundant functional groups on the cell surface offer a good environment for the self-assembly of Fe_3_O_4_@CS (Ding et al., 2015). The morphology of Fe_3_O_4_ particles, Fe_3_O_4_@CS and CtFC is shown in Figure 3. The imaging results demonstrated that the Fe3O4@CS was dispersed on the surface of the cells. The average particle size of 10–15 nm was calculated based on SEM images. EDS analysis detected carbon (64.64%), nitrogen (23.04%), oxygen (8.2%), and iron (4.12%). The TG-DSC curves (Figure 4) showed the thermal properties of the obtained Fe_3_O_4_ nanoparticles and Fe_3_O_4_@CS. Compared to the Fe_3_O_4_ particles, the TGA curve indicated that Fe_3_O_4_@CS went through three weight change processes under 800°C, which were related to the loss of residual water, followed by the decomposition and combustion of chitosan, respectively. These findings were in agreement with the DSC curve. The amount of chitosan bound to the Fe_3_O_4_ particles can be estimated on the basis of the percentage of weight loss in the TGA curve and the average mass content was 49.81% (Li et al., 2008). As can be seen in Figure 5, the hysteresis loops illustrated the superior saturation magnetization (Ms) of the prepared Fe_3_O_4_ particles, with 80 emu/g, which outperformed the Fe_3_O_4_ particles obtained by Luo et al. (2017), with an Ms value of 66.6 emu/g. The katabatic Ms value of Fe_3_O_4_@CS indicated that coating with chitosan blocked the Ms of pure Fe_3_O_4_ particles to a certain degree. In addition, the indistinguishable Ms value of Fe_3_O_4_@CS and CtFC may also be explained by the SEM findings that Fe_3_O_4_@CS was attached to the cell surface, so that the Ms was not affected. The XRD patterns of Fe_3_O_4_ nanoparticles, Fe_3_O_4_@CS and CtFC are depicted in Figure 6. All three samples showed the characteristic peaks of Fe_3_O_4_ at 30.1, 35.5, 43.1, 53.4, 57.0 and 62.6°, with their corresponding indices (220), (311), (400), (422), (511) and (440), which was highly matched with the trans-spinel structure of Fe_3_O_4_ MNPs (JCPDS, no. 65-3107) and confirmed that the Fe_3_O_4_ nanoparticles were well-crystallized. It also illustrated that the coating process of chitosan and Fe_3_O_4_@CS both did not bring about a phase change of Fe_3_O_4_. The average crystallite size of Fe_3_O_4_ calculated using the Scherrer equation was 10.9 nm, which was in good agreement with the size estimated from the SEM images. The mechanism driving the coating of cells by Fe_3_O_4_@CS was explored by analyzing the FTIR spectra (Figure 7). The characteristic peak at 579.31 cm^–1^, assigned to the Fe-O bending vibration, was present in all three samples, which was in agreement with the reported crystalline lattice of Fe_3_O_4_ (Zhang et al., 2010). New characteristic peaks at 1069.47 and 1071.63 cm^–1^ (C-O-C stretching vibration), 2908.14 cm^–1^ (stretching vibration of -CH_2_) and 3440.62 cm^–1^ (stretching vibrations of O-H and N-H bonds) were observed in the IR spectra of Fe_3_O_4_@CS and CtFC. These peaks can be explained by the primary layer of chitosan (tightly chemisorbed) on Fe_3_O_4_ nanoparticles. The interaction between the cells and Fe_3_O_4_@CS resulted in the displacement of the absorption peak in the fingerprint region (600–1800 cm^–1^) and a change of partial peak width, which may be related to hydrogen bonding and electrostatic repulsion (Li et al., 2009). Furthermore, all observed peaks of CtFC maintained a high degree of similarity with those of Fe_3_O_4_@CS, suggesting that the main structure of the Fe_3_O_4_@CS microspheres was not altered by surface coating the cells.

### Protective Effect of Fe_3_O_4_@CS on Cells Under Acid Stress

#### Growth Curve, Cell Viability, and Membrane Potential

**FIGURE 8:**
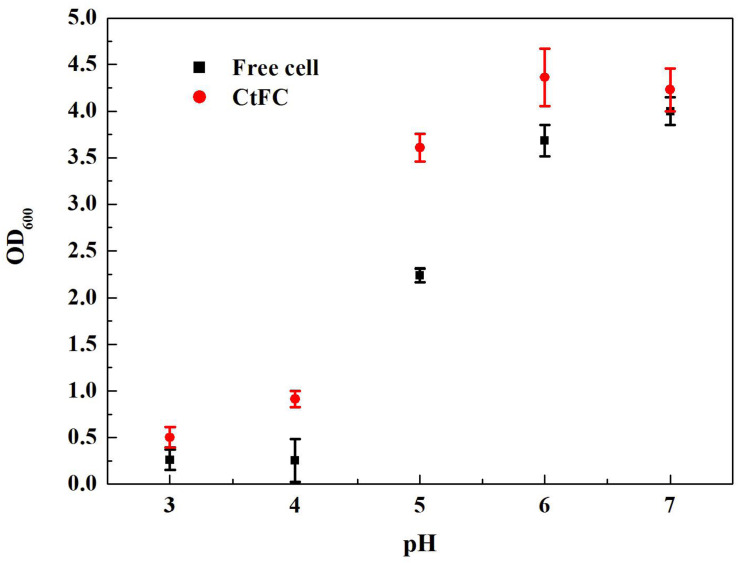
Effects of different pH on growth curves of free cells and CtFC.

**FIGURE 9:**
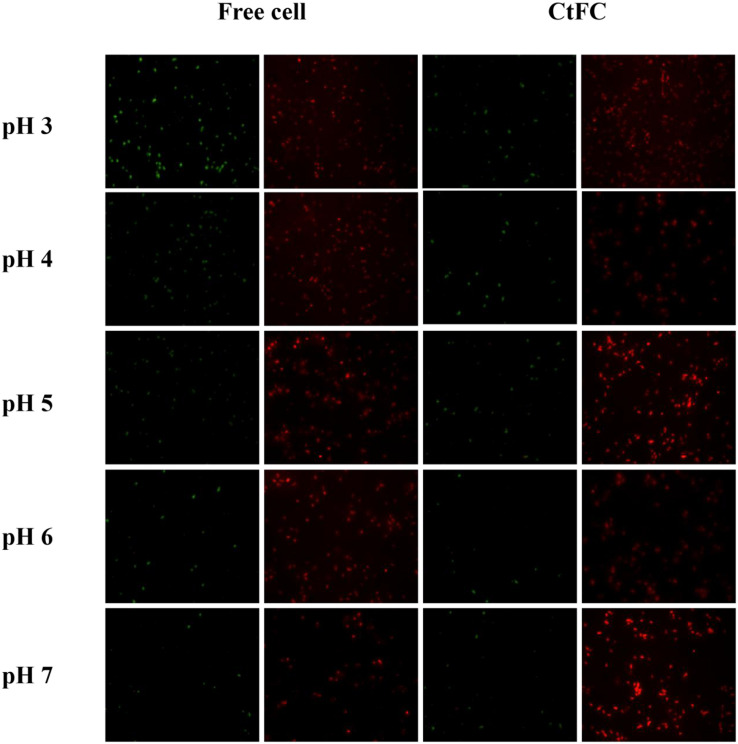
Cell viability and membrane potential detection of free cells and CtFC at different pH.

The effects of different pH values on the growth performance of free cells and CtFC were investigated after 12 h of anaerobic culture. As shown in Figure 8, CtFC exhibited obviously higher growth curve, with OD_600_ values from 0.50 at pH 3 to 4.23 at pH 7. At pH 5 there was a 60.7% increase of the OD_600_ value compared to free cells. Figure 9 showed the cell viability detected using the green fluorescent probe AnnexinV-FITC and mitochondrial membrane potential assessed using the red fluorescent probe Mito-Tracker Red CMXRos, for both free cells and CtFC grown at different pH values. With the decrease of pH, the numbers of green fluorescent free cells increased, indicating that the acidic environment was adverse for cell growth and had a negative effect on cell viability. More green fluorescent CtFC were also visualized. At the same time, the larger number of red fluorescent CtFC indicated a better stability of the membrane potential at different pH values compared to the free cells. Significant quantitative differences between free cells and CtFC were found for green fluorescence at pH 3, as well as red fluorescence at pH 6 and 7. These results suggested that Fe_3_O_4_@CS was efficacy precipitated as a coating shell, which protected *C. tyrobutyricum* from acid stress.

#### Oxidative and Cellular Damage

**FIGURE 10:**
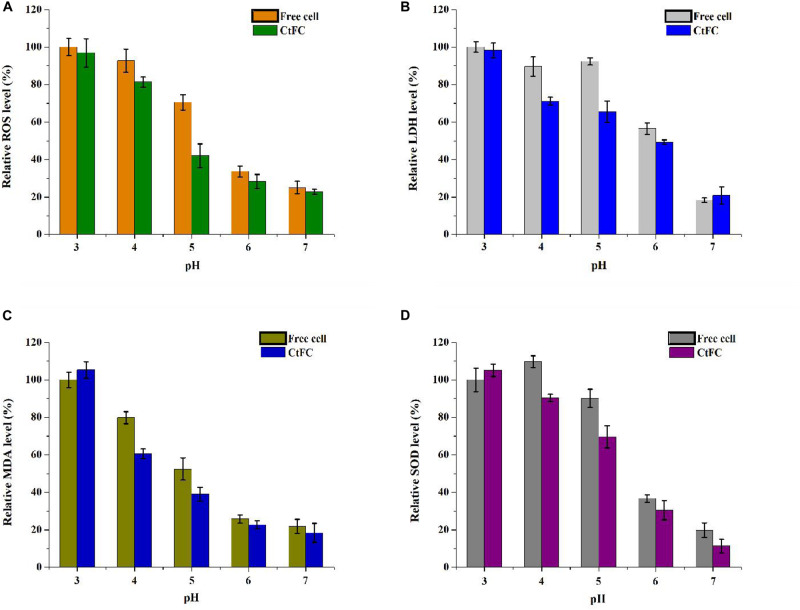
Relative ROS **(A)**, LDH **(B)**, MDA **(C)**, and SOD **(D)** levels in free cells and CtFC at different pH (3, 4, 5, 6, and 7) (*p* < 0.05).

Physiological indicators of free cells and CtFC were also measured to investigate the protective mechanism of Fe_3_O_4_@CS as a wrapping shell on the surface of the cells. As shown in Figure 10A, the ROS levels of CtFC were lower than those of free cells from pH 3 to 7, and were negatively correlated with the rise of pH in both groups. At low pH, the amount of ROS in the free cells and CtFC was high, indicating that the acid environment induced oxidative stress in *C. tyrobutyricum*. The amount of ROS in free cells was significantly higher than in CtFC at pH 4 and 5, while the difference was not as pronounced at pH 3. At pH 7, there were still significant ROS levels in free cells and CtFC, which was likely related to the intrinsic oxygen sensitivity of the anaerobic clostridia. Figure 10B revealed the changes in the amounts of LDH in free cells and CtFC exposed to different pH values. At pH 4 and 5, the relative LDH level in CtFC was significantly lower than that of free cells, especially at pH 5, which indicating that the shell formed by Fe_3_O_4_@CS protected the cell membrane from the acid attack. Interestingly, the amount of LDH in free cells at pH 5 was slightly higher than at pH 4, which reduced the negative correlation between LDH levels in free cells and the pH value. However, in a wide pH range of 3 to 7, the LDH levels in CtFC were negatively correlated. The content of MDA was shown in Figure 10C. It was clearly visible that the MDA level of CtFC gradually decreased as the environmental pH was lowered. Surprisingly, the amount of MDA at pH 3 was higher in CtFC than in free cells. The phenomenon could be explained that a highly acidic environment may induce the release of the Fe_3_O_4_@CS exoskeletons and the formation of naked Fe_3_O_4_ to some extent, while a handful of naked Fe_3_O_4_ left on the surface of the cells may bring mild cytotoxicity (Guo et al., 2017). The protection of the Fe_3_O_4_@CS shell thus greatly improved the acid tolerance, which was supported by the observation that the MDA level of CtFC was distinctly lower than that of free cells at pH 4 and 5. The SOD level was also measured to investigate the oxidation resistance induced by acid stress of free cells and CtFC cultured anaerobically for 12 h. As can be seen in Figure 10D, in a wide pH range of 3 to 7, the content of SOD in CtFC was negatively correlated. At pH 5, the amount of SOD in free cells was significantly higher than in CtFC, indicating that the oxidative stress response of free cells without the protection of the Fe_3_O_4_@CS coating was significantly activated. These results indicate that an overly acidic environment could induce the dissociation of weak acidic groups in polysaccharide components of the cell wall of *C. tyrobutyricum* and the surface proteins, which may lead to the shedding of Fe_3_O_4_@CS nanoparticles from the cell surfaces (Huang et al., 2018). As a result, *C. tyrobutyricum* with a Fe_3_O_4_@CS coating could gradually become revert to the form of free cells. This also explained to some extent why the ROS levels, MDA concentration, LDH content and SOD activity of free cells and CtFC were not significantly different at pH 3.

### *In situ* Repeated-Batch Fermentation of Butyric Acid Using CtFC

**FIGURE 11:**
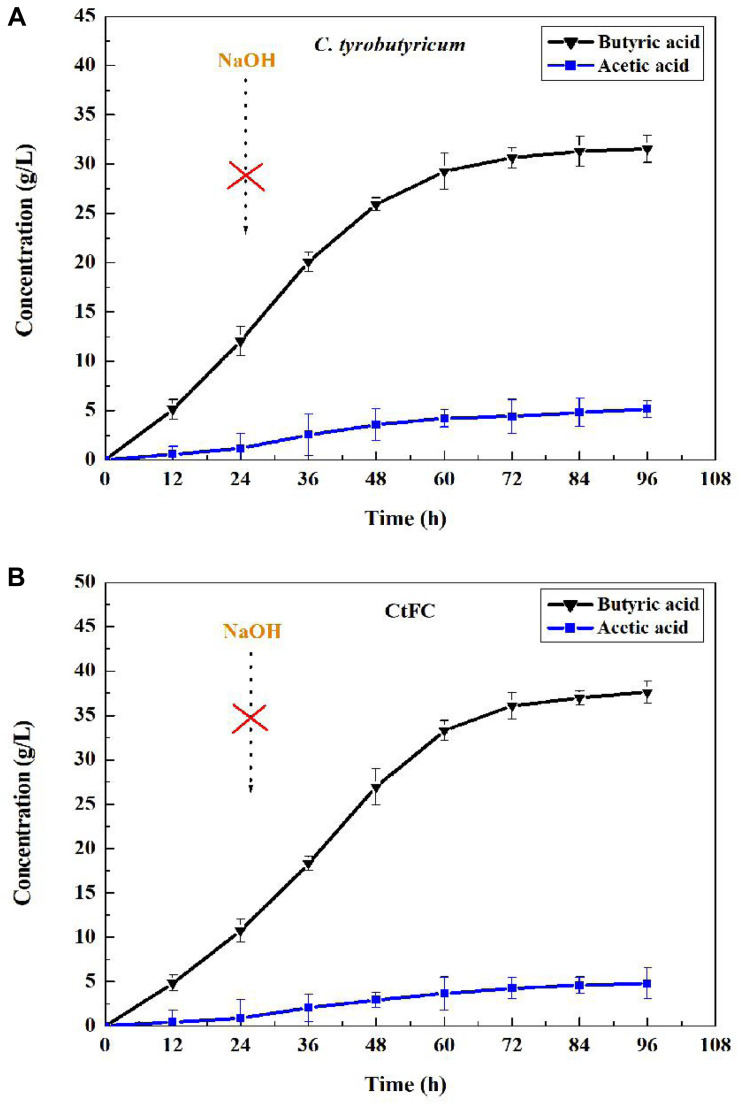
Batch fermentation of butyric acid in *C. tyrobutyricum* **(A)** and CtFC **(B)** without adding NaOH.

For microbiological butyric acid production, a mass of base (NaOH) is needed to maintain pH stable because of the excess acids accumulation resulting in the pH decrease of the fermentation broth (Wu et al., 2017). This Fe_3_O_4_@CS microspheres were proved to have the ability of acid protection, which may assist *C. tyrobutyricum* overcoming acid stress from fermentation environment. Based on this, batch fermentation of butyric acid between free cells and CtFC were investigated without adding NaOH. The results showed that pH decrease caused by the absence of NaOH led to the low concentration and yield of butyric acid in free cells, while had not significant effect in CtFC fermentation (Figure 11). As can been seen in Figure 11, the CtFC fermentation produced more butyric acid and reached a higher final concentration of 37.60 g/L, which was 19% higher than that from free-cell fermentation (37.60 vs. 31.56 g/L). The final butyric acid yield of 0.47 g/g was obtained with CtFC, which was as high as with free-cell *C. tyrobutyricum* under optimal pH (e.g., pH 6) via the addition of NaOH in traditional operations (Jiang et al., 2009). The above results suggested that the presence of Fe_3_O_4_@CS microspheres improved the defense ability of *C. tyrobutyricum* against the substrate toxicity and acidic environment during butyric acid fermentation. Moreover, avoiding the need for NaOH addition may improve the economics of industrial butyric acid production.

**FIGURE 12:**
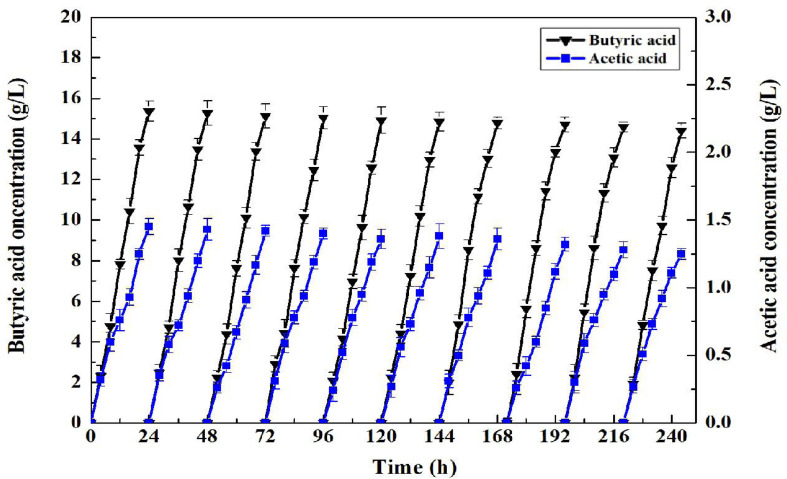
Repeated-batch fermentation of butyric acid in *C. tyrobutyricum* and CtFC. The original concentration of glucose in each batch was set at 32 g/L.

The superparamagnetism of Fe_3_O_4_ nano particles endowed enabled convenient separation and recycling of the whole-cell biocatalyst. Consequently, *C. tyrobutyricum* with the coating of Fe_3_O_4_@CS was both convenient and improved the accumulation of butyric acid in the fermentation process. To assess the superior properties of butyric acid production using CtFC, batch fermentations of 24 h were repeated 10 times. As presented in Figure 12, CtFC exhibited very good operational stability, with 15.36 g/L of butyric acid in the first batch and 14.40 g/L butyric acid in the 10^th^ batch. The butyric acid yield from glucose in all batch fermentations ranged from 0.48 to 0.45 g/g, and an average butyric acid yield of 0.46 g/g, which was higher than the 0.43 g/g average butyric acid yield reported by Huang et al. (2018). The result indicated there was no lag phase in all 10 batches. In addition, compared to free-cell fermentation, a higher butyrate/acetate ratio (11.89 vs. 6.12 g/g) was obtained in CtFC in repeated-batch fermentations, indicating some physiological modifications related to intracellular pH maintenance has been achieved in CtFC which can drive more metabolic flux toward butyric acid synthesis pathways (Huang et al., 2016, 2018).

## Conclusion

To our best knowledge, this is the first study in which an ordered and disperse acid-protective coat was generated by the self-assembly of Fe_3_O_4_@CS microspheres on the surface of *C. tyrobutyricum* cells. The optimal parameters for coating *C. tyrobutyricum* with Fe_3_O_4_@CS were determined by an orthogonal test, and were found to encompass a temperature of 37°C, a stirring speed of 80 rpm, and a mass ratio of Fe_3_O_4_@CS to DCW of 1:2. The superparamagnetism, thermostability, and subsize of Fe_3_O_4_@CS attached to the cell surface was determined visually and via physicochemical characterization. The analysis oxidative and cellular damage revealed higher levels of ROS, MDA, LDH, and SOD in free cells compared to the CtFC under acidic conditions, especially at pH 4 or 5, indicating the significant protective effect of the Fe_3_O_4_@CS microspheres. A higher butyric acid titer (37.60 vs. 31.56 g/L) was obtained in CtFC fermentation compared to free-cell fermentation without NaOH supplementation. Additionally, CtFC exhibited almost the same acid-producing activity when reused for 10 cycles with the butyric acid yield ranging from 0.48 to 0.45 g/g. These results suggested the potential of Fe_3_O_4_@CS microspheres in acid protection of *C. tyrobutyricum*. Based on this, the reusability and convenient separation of Fe_3_O_4_@CS microspheres provide the possibility for economical bio-production of butyric acid in large scale.

## Data Availability Statement

All data generated or analyzed in this study are available in this article.

## Author Contributions

TL performing the experiments and drafting of article. CJ assisted with the experiments. LZ analyzing the data. LJ designing the study. HH critical revision. All authors approved the manuscript for publication.

## Conflict of Interest

The authors declare that the research was conducted in the absence of any commercial or financial relationships that could be construed as a potential conflict of interest.
